# FAT1-associated membranous nephropathy after hematopoietic stem cell transplantation: a case report and review of the literature

**DOI:** 10.1007/s13730-026-01188-1

**Published:** 2026-09-30

**Authors:** Ryosuke Saiki, Kan Katayama, Yuto Matsui, Tomohiro Murata, Kaoru Dohi

**Affiliations:** Department of Cardiology and Nephrology, Mie University Graduate School of Medicine, 2- 174 Edobashi, Tsu, Mie 514-8507 Japan

**Keywords:** Case report, FAT1, Membranous nephropathy, Hematopoietic stem cell transplantation, Nephrotic syndrome

## Abstract

We report a 55-year-old man who developed nephrotic syndrome 52 months after allogeneic hematopoietic stem cell transplantation for acute lymphoblastic leukemia. He had chronic graft-versus-host disease controlled with low-dose prednisolone and tacrolimus. Asymptomatic proteinuria was detected five months before referral, followed by progressive edema, massive pleural effusion, and ascites. At presentation, laboratory testing showed profound hypoalbuminemia, mildly reduced kidney function, and nephrotic-range proteinuria. Kidney biopsy demonstrated membranous nephropathy, with periodic acid-methenamine silver staining showing spikes and bubbling of the glomerular basement membrane. Immunofluorescence showed granular capillary-wall immunoglobulin G staining with immunoglobulin G1 predominance over immunoglobulin G4, and protocadherin FAT1 immunofluorescence showed granular staining along the glomerular basement membrane. Prednisolone was increased from 5 to 30 milligrams per day while tacrolimus was continued, resulting in improved proteinuria, serum albumin, edema, and kidney function at five months without clinically significant infectious complications. We also reviewed 29 reported cases of protocadherin FAT1-associated membranous nephropathy after hematopoietic stem cell transplantation, including the present case. The review showed variable onset timing, substantial proteinuria at presentation, inconsistent immunoglobulin G4 positivity, and heterogeneous treatment with corticosteroids, rituximab, calcineurin inhibitors, and other regimens. Our case and review indicate that this disease can present late, cannot be excluded by non-immunoglobulin G4-predominant staining, and remains heterogeneous in diagnostic evaluation, treatment, and outcomes. Further accumulation of cases is needed to clarify the clinical spectrum and optimal management of this disease.

**Supplementary Information:**

The online version contains supplementary material available at https://doi.org/10.1007/s13730-026-01188-1.

## Introduction

Membranous nephropathy (MN) is the most frequently reported cause of nephrotic syndrome developing after hematopoietic stem cell transplantation (HSCT), accounting for 65.5% of adult post-HSCT nephrotic syndrome cases [1]. The classification of MN has evolved with the identification of multiple target antigens, and FAT1 has recently been identified as a novel target antigen specifically associated with MN developing after HSCT [2]. FAT1 is an atypical cadherin involved in cell adhesion, migration, and growth [3]. In the kidney, FAT1 is expressed in podocytes and contributes to the glomerular slit diaphragm [3, 4]. In reported post-HSCT FAT1-associated MN, FAT1 has been detected within glomerular immune deposits and anti-FAT1 IgG has been identified in biopsy eluates and serum, supporting an antibody-mediated mechanism [2]. However, the trigger for loss of immune tolerance to FAT1 after HSCT remains unclear.

Post-HSCT MN should not be regarded as a single antigen-defined disease. Although FAT1 has been identified as a major target antigen in this setting, PLA2R-positive, NELL1-positive, and antigen-unidentified cases have also been reported [2, 5, 6]. Target antigen identification has moved MN management toward individualized, serology-informed care. For instance, in PLA2R- and THSD7A-associated MN, antibody profiles can refine diagnosis, guide immunosuppressive treatment, and monitor relapse risk [7]. Although such an approach has not yet been established for FAT1-associated MN, it underscores why antigen-based classification may be clinically useful in post-HSCT MN.

Further accumulation and review of cases are needed to define the clinical spectrum of FAT1-associated MN after HSCT. Here, we report a case of FAT1 staining-positive MN in which asymptomatic proteinuria was first detected 52 months after allogeneic HSCT and nephrotic syndrome became clinically overt thereafter. We also review reported FAT1-associated post-HSCT MN cases, focusing on onset timing, IgG subclass patterns, diagnostic methods, treatment regimens, and outcomes.

## Case report

A 55-year-old man was referred to our department for a 2-month history of lower extremity edema. He had been diagnosed with acute lymphoblastic leukemia (ALL) 84 months prior to presentation and underwent HSCT 57 months prior to presentation, following chemotherapy. During the initial chemotherapy phase, portal vein thrombosis (PVT) and gastric varices were identified. However, anticoagulant therapy was withheld due to a high risk of bleeding. Following HSCT, the patient maintained hematological remission. Intermittent pruritus and episodic liver enzyme elevation suggested chronic graft-versus-host disease (cGVHD), and he had required temporary glucocorticoid dose increases on several prior occasions. However, during the 13 months preceding the onset of proteinuria, prednisolone (5 mg/day) and tacrolimus (1 mg/day) had been continued without dose reduction, discontinuation, or other treatment changes, and his cGVHD had remained clinically stable without evidence of reactivation. Five months prior to presentation, 52 months after HSCT, asymptomatic proteinuria (dipstick 2+) was detected. Four months prior to presentation, follow-up revealed asymptomatic esophageal varices, and edoxaban was initiated for PVT. Subsequently, he developed lower extremity edema two months prior to presentation, which prompted his referral to our department.

His past medical history was otherwise unremarkable except for an appendectomy at 15 years of age. Physical examination revealed severe edema accompanied by massive pleural effusion and ascites. His vital signs were as follows: blood pressure, 104/55 mmHg; heart rate, 63 beats/min; body temperature, 36.8 °C; and oxygen saturation, 98% while receiving 4 L/min of supplemental oxygen. Laboratory evaluation revealed profound hypoalbuminemia (0.8 g/dL) and mildly reduced kidney function (serum creatinine, 1.36 mg/dL; eGFR, 43.9 mL/min/1.73 m²). Urinalysis showed marked microscopic hematuria and granular casts. Proteinuria was in the nephrotic range (24-hour urinary protein, 8.96 g/day). Further workup was performed to determine the etiology of the nephrotic syndrome. Viral screening for hepatitis B and C was negative. Autoimmune screening, including antineutrophil cytoplasmic antibodies (ANCA) and antinuclear antibodies (ANA), was negative, and complement levels were within normal limits. Although an IgG-kappa monoclonal protein was detected in serum, the serum free light-chain ratio was normal at 1.19, and kappa and lambda staining in glomerular deposits was balanced. Additionally, thoracentesis and paracentesis yielded transudative fluids, which were negative for both bacterial cultures and malignant cells on cytology. No chronic or recurrent infection was clinically apparent.

**Fig. 1:**
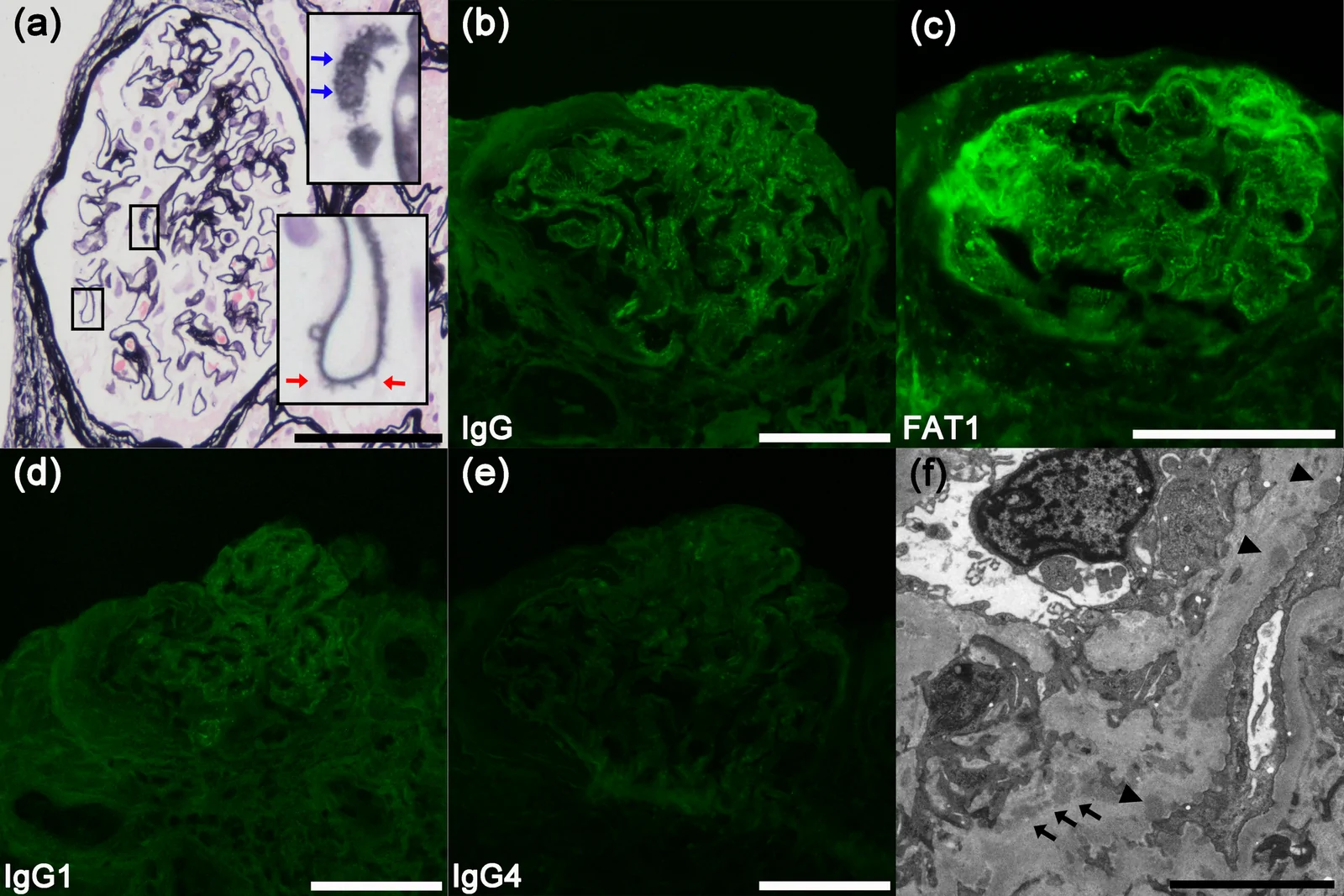
Kidney biopsy findings. **a** Periodic acid-methenamine silver staining showed spikes (red arrows) and a bubbling appearance (blue arrow). Scale bar = 100 μm. Enlarged views of representative lesions are shown in the insets. **b** Immunofluorescence staining for IgG showed granular capillary-wall positivity. Scale bar = 100 μm. **c** Immunofluorescence staining for protocadherin FAT1 showed granular staining along the glomerular basement membrane. Scale bar = 100 μm. **d, e** IgG subclass staining showed IgG1 predominance, with strong IgG1 staining (**d**) and weak IgG4 staining (**e**). Scale bars = 100 μm. **f** Electron microscopy showed subepithelial electron-dense deposits (arrowheads), partial foot-process effacement, and additional mesangial or paramesangial electron-dense deposits (arrows), consistent with stage II membranous nephropathy. Scale bar = 5 μm. Abbreviations: FAT1, protocadherin FAT1; IgG, immunoglobulin G; PAM, periodic acid-methenamine silver

A kidney biopsy was performed. Periodic acid-methenamine silver (PAM) staining showed spikes and a bubbling appearance in the glomerular basement membrane (Fig. 1a). Direct fast scarlet staining was negative, including under polarized light. Immunofluorescence demonstrated granular capillary-wall positivity for immunoglobulin G (IgG) (Fig. 1b). A complete set of routine immunofluorescence findings is provided in Online Resource 1. IgA, C3, C1q, and fibrinogen showed mild glomerular staining; IgM was negative, and focal punctate signals were considered nonspecific; and kappa and lambda light-chain staining showed comparable intensity and distribution. Given the clinical context of MN after allogeneic HSCT, immunofluorescence staining for protocadherin FAT1 was performed and showed granular staining along the glomerular basement membrane (Fig. 1c). IgG subclass analysis revealed IgG1 predominance over IgG4 (Fig. 1d, e). Electron microscopy findings were consistent with stage II membranous nephropathy and additionally revealed mesangial electron-dense deposits (Fig. 1f).

In the post-HSCT context, the clinicopathologic findings and FAT1 staining supported FAT1-associated MN. Regarding management, renin-angiotensin system blockade was not initiated due to the patient’s baseline hypotension. Supportive therapy with diuretics was administered to manage the severe edema and serosal effusions. Prednisolone was increased to 30 mg/day (approximately 0.5 mg/kg/day), while tacrolimus 1 mg/day was continued. At 5 months after treatment intensification, prednisolone had been tapered to 12.5 mg/day, and tacrolimus remained 1 mg/day. Serum creatinine was 1.25 mg/dL (eGFR, 47.9 mL/min/1.73 m²), the urine protein-to-creatinine ratio was 0.60 g/gCr, serum albumin was 3.1 g/dL, edema had improved, and no clinically significant infectious complications had occurred.

## Discussion

This case and the literature review illustrate the heterogeneity of FAT1-associated MN after HSCT in timing, IgG subclass patterns, diagnostic methods, treatment regimens, and outcomes.

The original report by Sethi et al. identified 14 FAT1-associated MN cases after allogeneic HSCT using laser microdissection and mass spectrometry, immunohistochemistry, confocal immunofluorescence, and anti-FAT1 IgG detection in biopsy eluates or serum [2]. Subsequent reports added further cases and provided additional information on clinical course, treatment, and outcomes [5, 6, 8]. Including the present case, Table 1 summarizes 29 FAT1-associated post-HSCT MN cases. The mean age was 55.0 years, and 19 patients were male (65.5%). Among 16 cases with individually reported timing, the mean interval from HSCT to MN onset was 29.4 months (median, 24 months; range, 10–57 months); Nackenhorst et al. reported a cohort median of 2.3 years with an interquartile range of 1.3–4.4 years, but individual timing data were not available [5]. Proteinuria at onset was substantial, with a mean of 7.91 g/day among 17 cases reported in g/day and 7.99 g/gCr among 12 cases reported in g/gCr. Among 18 cases with IgG4 data, 7 (38.9%) were IgG4-positive or IgG4-dominant. The clinicopathological significance of IgG subclass variation in FAT1-associated MN remains uncertain. Treatment was reported in 20 cases: corticosteroids were used in 13 (65.0%), rituximab in 10 (50.0%), calcineurin inhibitors in 11 (55.0%), cyclophosphamide in 2 (10.0%), plasma exchange in 1 (5.0%), and no immunosuppression in 1 (5.0%). These data indicate that FAT1-associated MN after HSCT is recognized as an antigen-defined entity, but its clinical spectrum and optimal treatment remain unclear.

**Table 1:** Clinicopathological characteristics, treatment, and outcomes of FAT1-associated membranous nephropathy after hematopoietic stem cell transplantation: previously reported cases and the present case

References	Case No.	Age	Sex	Time from HSCT (months)	PU (at onset)	Cr (mg/dL)	ALb (g/dL)	IgG4^2^	Electron microscopy	Treatment regimen	Proteinuria response	Survival
Sethi et al. [2]	1	57	F	18	3.0 g/day	NA	NA	+	Subepithelial deposits	NA	NA	Deceased (3 mo after diagnosis, Cause NA)
Sethi et al. [2]	2	69	F	48	3.8 g/day	1.20	NA	NA	Subepithelial + TBM deposits	RTX, Steroids	NA	Deceased (3 mo after diagnosis, Cause; Congestive heart failure)
Sethi et al. [2]	3	69	F	24	3.5 g/day	2	NA	+	Subepithelial + TBM deposits	RTX	0.3 (24 mo)	Alive (24 mo follow-up)
Sethi et al. [2]	4	54	F	36	17 g/day	2.60	NA	NA	Subepithelial + TBM deposits	NA	0.2 (64 mo)	Alive (64 mo follow-up)
Sethi et al. [2]	5	54	M	NA	22 g/day	1.40	NA	NA	Subepithelial deposits	NA	NA	Deceased (22 mo after diagnosis, Cause; NA)
Sethi et al. [2]	6	53	M	36	4.4 g/day	1.30	NA	NA	Subepithelial deposits	NA	0.3 (41 mo)	Alive (41 mo follow-up)
Sethi et al. [2]	7	63	M	24	7 g/day	1	NA	+	Subepithelial deposits	NA	1.1 (83 mo)	Alive (83 mo follow-up)
Sethi et al. [2]	8	42	F	24	15 g/day	1.10	NA	NA	Subepithelial deposits	NA	0.3 (67 mo)	Alive (67 mo follow-up)
Sethi et al. [2]	9	71	M	24	8 g/day	1.20	NA	+	Subepithelial deposits	NA	NA	Deceased (8 mo after diagnosis, Cause; NA)
Sethi et al. [2]	10	68	M	24	5.6 g/day	1.10	NA	+	Subepithelial deposits	NA	7.8 (96 mo)	Alive (96 mo follow-up)
Sethi et al. [2]	11	69	M	48	9 g/day	1.50	NA	+	Subepithelial deposits	NA	NA	Alive (no date)
Sethi et al. [2]	12	51	M	24	2 g/day	0.70	NA	NA	Subepithelial deposits	CyA, Steroids	0.3 (156 mo)	Deceased (156 mo after diagnosis, Cause; Sepsis)
Sethi et al. [2]	13	62	M	24	4 g/day	1.50	NA	NA	Subepithelial deposits	Steroids	0.3 (96 mo)	Alive (96 mo follow-up)
Sethi et al. [2]	14	63	M	24	5.4 g/day	1.10	NA	NA	Subepithelial deposits	CyA, Steroids	1.2 (4 mo)	Deceased (4 mo after diagnosis, Cause; Sepsis)
Nackenhorst et al. [5]	1	59	F	Median: 2.3 years (IQR: 1.3–4.4)^1^	1.5 g/gCr	0.78	NA	-	Cohort finding (*n* = 9 tested): Subepithelial deposits (all); Additional Subendothelial deposits (1 case)^1^	No Immunosuppression	0.1 (12mo), 0 (36mo)	Alive (182 mo follow-up)
Nackenhorst et al. [5]	2	39	F		3.9 g/gCr	1.19	NA	-		Steroids	0.3 (36mo)	Alive (108 mo follow-up)
Nackenhorst et al. [5]	3	63	F		9 g/gCr	1.30	NA	-		RTX	PR^3^	Alive (8 mo follow-up)
Nackenhorst et al. [5]	5	55	M		6.5 g/gCr	0.80	NA	-		RTX, TAC	2.0 (12mo), 3.4 (36mo), 7 (60mo)	Deceased (63 mo after diagnosis, Cause; Cerebral edema)
Nackenhorst et al. [5]	7	26	M		8 g/gCr	0.68	NA	-		CyA, CYC, RTX, Steroids	0.2 (12mo), < 0.1 (36mo)	Alive (161 mo follow-up)
Nackenhorst et al. [5]	8	50	M		8.3 g/gCr	1.24	NA	-		CyA, Steroids	3.0 (12mo), 0.6 (36mo), 0.3 (60mo)	Alive (60 mo follow-up)
Nackenhorst et al. [5]	10	51	F		4.7 g/gCr	0.87	NA	NA		CyA, CYC, PE, Steroids, TAC	Non-Responder^2^	Deceased (8 mo after diagnosis, Cause; NA)
Nackenhorst et al. [5]	11	49	M		4.4 g/gCr	0.80	NA	-		CyA, RTX	2.5 (12mo), 0.1 (36mo), 0.1(60mo)	Alive (159 mo follow-up)
Nackenhorst et al. [5]	12	60	M		9 g/gCr	1.39	NA	+		RTX	2.8 (12mo)	Alive (25mo follow-up)
Nackenhorst et al. [5]	13	28	M		19 g/gCr	1.30	NA	-		RTX, Steroids	1.8 (12mo)	Alive (108 mo follow-up)
Nackenhorst et al. [5]	14	62	M		18 g/gCr	1.36	NA	-		RTX	NA	Alive (2 mo follow-up)
Nackenhorst et al. [5]	15	57	M		3.6 g/gCr	1.20	NA	-		CyA, RTX, Steroids	6.4 (12mo)	Alive (10 mo follow-up)
Bosnić Kovačić et al. [6]	1	41	F	26	11.8 g/day	1.17	NA	NA	Subepithelial deposits	CyA, Steroids	0.2 (no date)	Alive (147 mo follow-up)
Bosnić Kovačić et al. [6]	2	56	M	10	4 g/day	0.92	1.80	NA	Subepithelial deposits	CyA, Steroids	0.1 (no date)	Alive (78 mo follow-up)
Present Case	1	55	M	57	8.96 g/day	1.36	0.80	-	Subepithelial + paramesangial deposits	Steroids, TAC	0.60 (5mo)	Alive (5 mo follow-up)

Alb, Serum albumin; Cr, Creatinine; CyA, Cyclosporin A; CYC, Cyclophosphamide; F, Female; HSCT, Hematopoietic stem cell transplantation; IgG, Immunoglobulin G; M, Male; mo, months; PE, plasma exchange; PU, Proteinuria; RTX, Rituximab; TAC, Tacrolimus; TBM, Tubular basement membrane

1. Individual data were not provided

2. +: IgG4-positive and dominant; -: otherwise

3. Definitions per Nackenhorst et al. [5]: CR (Complete Remission): >90% reduction of initial albuminuria or < 0.5 g/day. PR (Partial Remission): >50% reduction of initial albuminuria

The timing in the present case was among the later reported presentations, with asymptomatic proteinuria first documented 52 months after HSCT. Potential mechanisms include alloimmune dysregulation during immune reconstitution, cGVHD-related autoimmunity, and glomerular injury exposing podocyte-associated FAT1 to immune recognition [2, 5]. In previous cases, nephrotic syndrome developed after withdrawal or reduction of GVHD prophylaxis or immunosuppressive therapy, suggesting that immune reactivation may contribute to disease onset [6, 8]. Despite clinically stable cGVHD and more than 1 year of unchanged low-dose prednisolone and tacrolimus therapy, proteinuria developed in this patient. Because the tacrolimus trough level immediately before onset was unavailable, inadequate drug exposure cannot be excluded. The level of immunosuppression required to prevent FAT1-associated MN remains unknown. Therefore, persistent proteinuria after HSCT should prompt evaluation for MN, including FAT1 staining.

The combination of IgG1 predominance and mild glomerular C1q and C3 deposition may suggest local classical complement pathway activation because IgG1 can bind C1q, whereas IgG4 has limited C1q-binding capacity. However, routine immunofluorescence demonstrates complement deposition and cannot determine the initiating pathway; pathway-specific assays were not performed in our case [9]. In a primary MN cohort, glomerular C1q deposition was not associated with treatment response or kidney outcomes [10], but C1q deposition has clinical significance in FAT1-associated MN remains unknown.

The antigen-based diagnosis has limitations. Although granular glomerular FAT1 staining in the clinical context of post-HSCT MN supported an interpretation compatible with FAT1-associated MN, PLA2R, THSD7A, and NELL1 were not evaluated in the present case. Therefore, dual antigen-positive forms of MN were not formally excluded. In addition, circulating anti-FAT1 antibodies were not measured, precluding serological confirmation of a patient-specific anti-FAT1 immune response. Previous FAT1-positive post-HSCT MN cases in which other antigens were evaluated were generally negative for known MN antigens, but these findings cannot establish antigen negativity in the present patient. Thus, FAT1 cannot be regarded as the definitively proven exclusive pathogenic antigen in this case. Monoclonal gammopathy-related MN cannot be completely excluded because an IgG-kappa monoclonal protein was detected. However, a previous study reported M proteins in 31 of 60 bone marrow transplant recipients (52.0%) [11]. In the present case, balanced glomerular kappa/lambda staining and a normal serum free light-chain ratio made monoclonal gammopathy-related MN less likely.

Treatment for FAT1-associated MN after HSCT remains undefined, and reported outcomes vary [2, 5, 6]. In the present case, proteinuria improved after prednisolone was increased while low-dose tacrolimus was continued. However, a single case is insufficient to determine the optimal treatment strategy. Further accumulation of cases is required to clarify the appropriate choice and intensity of therapy according to nephrotic severity, cGVHD activity, baseline immunosuppression, renal function, and infectious risk.

In conclusion, the present case and literature review show that FAT1-associated MN after HSCT remains clinically heterogeneous in timing, diagnostic evaluation, treatment, and outcomes. This case suggests that persistent proteinuria in long-term HSCT survivors should prompt evaluation for MN, including assessment of target antigens such as FAT1, even when it develops several years after HSCT during stable maintenance immunosuppression. Further accumulation of cases is needed to clarify the clinical spectrum and optimal management of this disease.

## Supplementary Information

Below is the link to the electronic supplementary material.

Supplementary Material 1 Immunofluorescence staining of the kidney biopsy specimen showed (a) granular capillary-wall positivity for immunoglobulin G (IgG) and (b) mild positivity for immunoglobulin A (IgA). (c) IgM was negative; focal punctate signals were considered nonspecific. (d) Complement component 3 (C3) and (e) complement component 1q (C1q) showed mild glomerular positivity, and (f) fibrinogen showed mild positivity. (g, h) κ and λ light chains showed granular capillary-wall staining with comparable intensity and distribution. (i–l) IgG subclass staining showed predominant IgG1 positivity, with weaker staining for IgG2, IgG3, and IgG4. Scale bars = 100 μm.

## Data Availability

The datasets used and/or analyzed during the current study are available from the corresponding author upon reasonable request.
